# Managing Neonatal Varicella: A Case Series Highlighting the Effectiveness of Antiviral Therapy Without IVIG

**DOI:** 10.3390/children12081081

**Published:** 2025-08-18

**Authors:** Filla Reviyani Suryaningrat, Sindy Irenewati, Devatri Hudayari, Fiva Aprilia Kadi, Aris Primadi, Tetty Yuniati

**Affiliations:** 1 Department of Child Health, Faculty of Medicine, Universitas Padjajaran, Dr Hasan Sadikin General Hospital, Bandung 40161, West Java, Indonesia; 2 Faculty of Medicine, Universitas Diponegoro, Semarang 50244, Central Java, Indonesia

**Keywords:** chicken pox, neonatal varicella syndrome, neonates, therapeutic management

## Abstract

**Background:** Chicken pox is a rare but serious condition in neonates—often regarded as a common childhood illness with mild symptoms—yet it can lead to severe complications, especially in the perinatal period. Neonatal varicella may present with fever occurring within the first 5–10 days of life, followed by a generalized vesicular eruption. The syndrome is uncommon, largely due to the widespread immunity in women of childbearing age, acquired through prior chicken pox infection or varicella immunization. However in Indonesia, a developing country without a national mandatory varicella vaccination program, the disease burden remains significant, and cases of neonatal varicella are still encountered. Neonates are at high risk of severe varicella infection, which, if untreated, has a reported mortality rate of up to 30%. Although varicella is rare in neonates, there are limited studies that have reported it. This study highlights the clinical presentations upon admission, diagnostic investigations, therapeutic management strategies, and potential complications of neonatal varicella. **Methods:** This study presents two cases of neonatal varicella that were managed at Hasan Sadikin General Hospital in West Java, Indonesia. Each patient underwent a clinical assessment and diagnostic evaluation upon arrival, followed by therapeutic management strategies, including the management of any complications that emerged during treatment. **Results:** The two cases of neonates presented with classic clinical features of neonatal varicella, including a generalized vesicular rash followed by fever within the first 10 to 12 days of life, without dermatological lesions or congenital malformations at birth. In both cases, maternal chicken pox developed within the first few days postpartum, suggesting postnatal transmission as the likely source of infection. Complications observed included respiratory failure and pneumonia, requiring respiratory support. However, both neonates recovered successfully without the administration of IVIG. **Conclusions:** Early initiation of antiviral therapy, timely administration of antibiotics, comprehensive supportive care, and monitoring for potential complications play a crucial role in managing neonatal varicella, even in the absence of IVIG.

## 1. Introduction

Varicella, commonly known as chicken pox, is caused by a primary infection with the varicella-zoster virus (VZV)**.** Without widespread vaccination, this highly contagious disease can affect the majority of individuals by mid-adulthood [1]. While varicella is generally considered a benign childhood illness, it can pose significant health risks in certain populations, particularly in neonates [2].

Neonatal varicella occurs when maternal infection develops during the last three weeks of pregnancy [1]. The clinical presentation and severity in the newborn largely depend on the timing of maternal infection relative to pregnancy. Infants are most at risk of severe disease if born from 5 days before to 2 days after onset of the maternal varicella rash [3]. Neonates with varicella often develop symptoms within the first 5–10 days of life, presenting with fever and a generalized vesicular rash. Without appropriate treatment, the condition can lead to severe illness, with a reported mortality rate of up to 30% [4].

The burden of disease is particularly relevant in developing countries like Indonesia, where a national mandatory varicella vaccination program is not in place. Consequently, neonatal varicella cases continue to be reported in clinical settings; however, very few studies have documented them. This study presents two cases of neonatal varicella treated at Hasan Sadikin General Hospital, West Java, Indonesia. It highlights clinical presentations, diagnostic investigations, therapeutic strategies, and potential complications, emphasizing the importance of early recognition and appropriate management of this condition.

## 2. Methods

We performed a prospective observational case series at Dr. Hasan Sadikin General Hospital, Bandung—the tertiary referral center for the West Java region of Indonesia—from 1 July to 31 December 2024, enrolling all consecutively admitted neonates aged ≤28 days with a clinically confirmed diagnosis of varicella and excluding those with major congenital anomalies.

Each infant underwent a clinical assessment and diagnostic evaluation upon arrival, followed by therapeutic management strategies, including the management of any complications that emerged during treatment. Demographic data, perinatal and maternal histories, dates of rash onset and admission, interventions, complications, length of stay, and survival to discharge were recorded prospectively. The study protocol received approval from the Research Ethics Committee of Dr Hasan Sadikin General Hospital (Approval No. 19852012020122007, 10 February 2025), and written informed consent for the study was obtained from each participant’s parent or legal guardian before enrolment.

## 3. Clinical Cases

### 3.1. Patient 1

A female baby was born to a first gravida mother at full term by normal vaginal delivery in a secondary care hospital. The mother had developed fever with vesicular skin eruptions and pruritus on the face, trunk, and limb, suggestive of chicken pox, at the time of delivery. Prior to delivery, the mother had no reported problems during pregnancy, such as hypertension, diabetes mellitus, abnormal vaginal discharge, or premature rupture of the membrane.

The baby cried soon after birth and had no dermatological lesions. Her birth weight was 2.7 kg, length 48 cm, and head circumference 34 cm, all of which were within the normal range for a term baby. There were no apparent congenital malformations. General examinations did not reveal any abnormalities.

**Figure 1:**
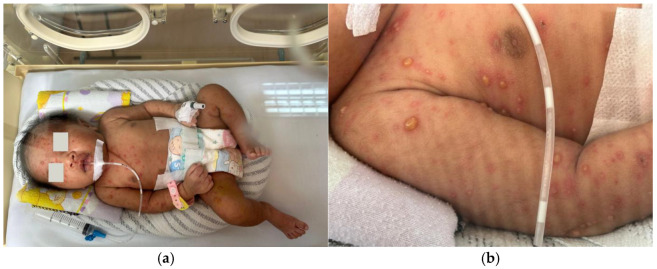
(**a**) Neonate presenting with lesions; (**b**) characteristic vesicular lesions.

On the fourteenth postnatal day, the patient was brought to Hasan Sadikin General Hospital presenting with multiple form lesions including discrete, shiny vesicular eruptions containing clear fluid and pus, measuring 0.5 cm × 0.5 cm × 0.1 cm and 0.6 cm × 0.8 cm × 0.2 cm, with well-defined borders. They were surrounded by erythema and crusts. These lesions were observed on the face, trunk, and limbs (Figure 1a,b). Her palms and soles were spared. The initial symptoms began two days prior, with vesicular eruptions appearing on the face, followed by fever one day prior to admission. There were no reported episodes of shortness of breath, seizures, or altered consciousness. No other household members reported similar symptoms, and no nearby neighbors showed similar signs. Based on the maternal history typical of chicken pox and the nature of rash on the baby, a diagnosis of neonatal varicella was made.

**Figure 2:**
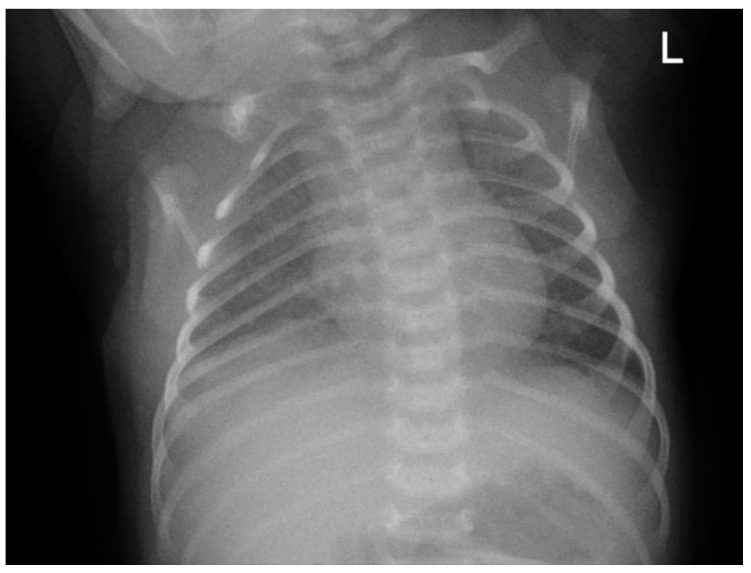
Anteroposterior (AP) chest X-ray on day 1 of admission to the emergency department (ED) showing normal cardiac and pulmonary structures with no abnormalities detected. “L”: an anatomical marker indicating the left side.

The patient was initially taken to a midwife and treated with a topical ointment, but the condition persisted. The patient was then brought to the secondary hospital where they were born and treated in the emergency department. As part of the initial therapy, the patient received intravenous ampicillin–sulbactam and gentamicin. The patient was subsequently referred to Hasan Sadikin General Hospital for further management (see Figure 2 for baseline chest X-ray taken on day 1 at the emergency department).

The baby was admitted to a neonatal high-care unit in isolation to prevent transmission. Initial and follow-up blood laboratory findings are summarized in Table 1. Fluid requirements were calculated at 150 mL/kg/day, consisting of expressed breast milk (EBM). The patient was started on intravenous acyclovir every 8 h for 7 days, while continuing ampicillin–sulbactam every 6 h and gentamicin every 24 h for 7 days.

**Figure 3:**
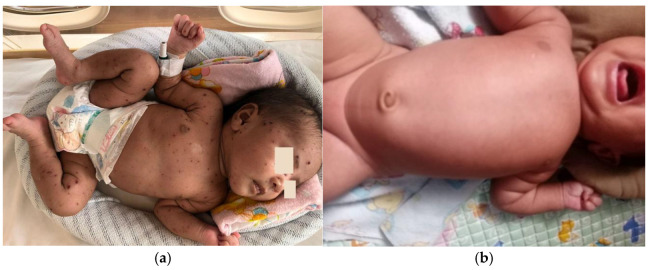
(**a**) Skin lesions after 7 days of therapy; (**b**) complete recovery with no remaining skin lesions or rashes resolved by day 10 after hospital discharge.

The patient was discharged on the eighth day of hospitalization in stable condition, with dried vesicular lesions and no new lesions observed (Figure 3a). There was no fever or respiratory distress. The patient was prescribed topical gentamicin ointment for continued skin lesion management at home and was advised to continue breastfeeding. All rashes disappeared by day 19 after the initial vesicular eruption (Figure 3b), and the recovery was complete and uneventful without the use of IVIG therapy.

### 3.2. Patient 2

A male baby was born to a second gravida mother at full term by normal vaginal delivery assisted by a midwife at a community health center. The mother had developed fever with vesicular skin eruptions and pruritus on the face, trunk, and limbs, suggestive of chicken pox three days after delivery. Prior to delivery, the mother had no reported problems during pregnancy, such as hypertension, diabetes mellitus, abnormal vaginal discharge, or premature rupture of the membrane.

The baby cried soon after birth and had no dermatological lesions. His birth weight was 3.0 kg, length 49 cm, and head circumference 32 cm, all of which were within normal range for a term baby. There were no apparent congenital malformations. General examinations did not reveal any abnormalities.

**Figure 4:**
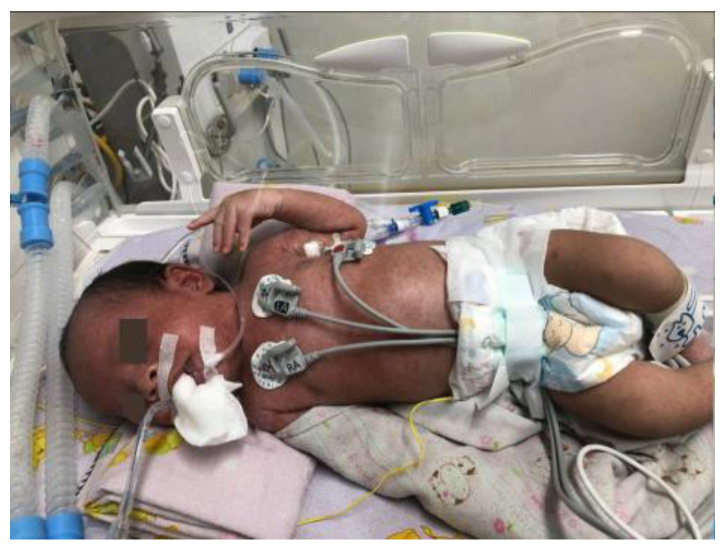
Neonate presenting with lesions on the first day of hospitalization.

On the twelfth postnatal day, the patient was brought to Hasan Sadikin General Hospital presenting with shortness of breath along with discrete, shiny vesicular eruptions measuring 0.3 × 0.4 × 0.1 cm and 0.5 × 0.5 × 0.2 cm. The eruptions contained clear fluid and were surrounded by erythema and crusts. These lesions were observed on the face, trunk, and limbs (Figure 4). His palms and soles were spared. The initial symptoms began three days prior, with vesicular eruptions appearing on the face, followed by fever, beginning two days prior. The condition worsened as the lesions spread, along with shortness of breath that with shortness of breath that started one day before admission to Hasan Sadikin General Hospital. The patient appeared lethargic and showed poor feeding. There were no reported episodes of seizures or altered consciousness. No other household members reported similar symptoms, and no nearby neighbors showed similar signs. Based on the maternal history typical of chicken pox and the nature of rash on the baby, a diagnosis of neonatal varicella was made.

**Figure 5:**
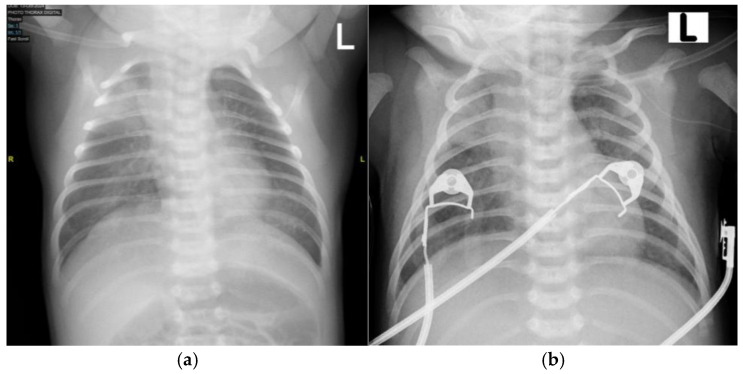
Anteroposterior (AP) chest X-ray showing (**a**) bilateral pneumonia on admission to the emergency department (ED) and (**b**) improvement in pneumonia on day 9 of hospitalization before extubation. “L”: an anatomical marker indicating the left side.

Upon initial examination, the patient exhibited respiratory distress, with grunting, subcostal chest retractions, and an SpO_2_ of 81% on room air. Oxygen supplementation was initiated using continuous positive airway pressure (CPAP), resulting in an improved oxygen saturation of 98%. A chest X-ray was performed, revealing pneumonia (Figure 5a). However, during monitoring, the patient’s respiratory distress worsened, with a decline in oxygen saturation and episodes of periodic apnea, necessitating intubation. The patient was subsequently transferred to the NICU for further respiratory support. Initial and follow-up blood laboratory findings are summarized in Table 2.

Fluid requirements were calculated at 150 mL/kg/day, consisting of total parenteral nutrition (TPN) and expressed breast milk (EBM). The patient was started on intravenous acyclovir every 8 h, ampicillin–sulbactam every 12 h, and gentamicin every 24 h.

On day 4 of hospitalization, the patient’s condition deteriorated, with worsening respiratory distress. Due to clinical worsening, ampicillin–sulbactam was discontinued, and cefotaxime was initiated every 6 h. The patient also received symptomatic therapy, including antipyretics as needed for fever and nebulized inhaled corticosteroids three times daily. Acyclovir was continued for a total of 8 days, while gentamicin was administered for 6 days.

By day 9 in the NICU, the patient’s general condition had stabilized, and extubation was performed (Figure 5b). On day 10 of hospitalization, the patient was transferred to the neonatal high-care unit (NHCU) for continued monitoring and supportive care. The patient remained in the NHCU for an additional 5 days, during which antibiotic therapy was continued, with cefotaxime given for a total of 12 days.

**Figure 6:**
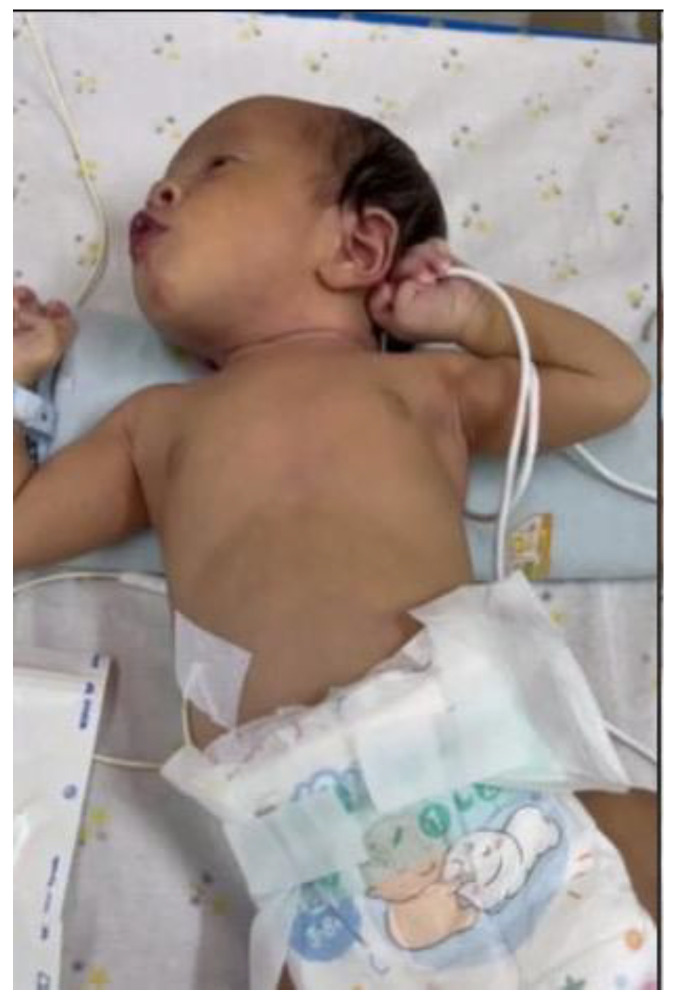
Complete recovery with no remaining skin lesions or rashes, resolving on day 14 of hospitalization.

All rashes disappeared by day 17 after the initial vesicular eruption, and recovery was complete and uneventful without the use of IVIG therapy (Figure 6). The patient was discharged on day 15 of hospitalization in stable condition, with no remaining skin rashes. There was no fever or respiratory distress.

## 4. Discussion

Varicella, also known as chicken pox, is a primary infection caused by the varicella-zoster virus (VZV) or human herpesvirus 3. This virus belongs to the *alphaherpesvirus* subfamily and carries a double-stranded DNA genome. VZV exclusively infects humans and has no known animal reservoir. The incubation period for varicella ranges from 10 to 21 days [5]. Varicella can affect all age groups, with the highest incidence in children under 10 years old, peaking between ages 5 and 9. Widespread varicella vaccination has significantly changed the epidemiology of the disease, leading to lower incidence rates in developed countries compared to developing countries without national mandatory vaccination programs [6]. The varicella-zoster virus (VZV) vaccine, a live attenuated vaccine, is included in the World Health Organization’s Essential Medicines for Children list, providing 98% protection against severe disease with a two-dose regimen [4]. However in Indonesia, a developing country without a national mandatory varicella vaccination program, varicella remains a concern, including in pregnant women, where the incidence is estimated at 1 to 5 cases per 10,000 pregnancies [4,7].

The clinical manifestations of neonatal varicella depend on the timing of exposure to the varicella-zoster virus (VZV). Congenital varicella syndrome (CVS) or fetal varicella syndrome (FVS) is a relatively rare condition in which affected infants present with distinct abnormalities at birth due to maternal varicella infection occurring within the first 20 weeks of gestation. The severity and range of associated symptoms and physical findings can vary significantly, depending on the timing of fetal exposure to VZV. Generally, the earlier the infection occurs during pregnancy, the more severe the resulting abnormalities [2]. The clinical manifestations of CVS are shown in Table 3.

On the other hand, neonatal varicella occurs when a pregnant woman contracts chicken pox during the last three weeks of pregnancy or within the first few days postpartum. The disease typically manifests in the newborn within the first 10 to 12 days of life, without features of congenital syndrome or structural abnormalities [8]. If the onset of maternal disease occurs between 5 days before delivery and 2 days postpartum, the risk of neonatal transmission is high (up to 50%), with a significant associated mortality rate of up to 30% [7]. These infants are typically exposed to high viral loads but lack the opportunity to acquire maternal protective antibodies. As a result, 20–50% of these neonates develop life-threatening disseminated disease between postnatal days 5–10, which may lead to severe complications such as varicella pneumonia, hepatitis, or meningoencephalitis [3]. In contrast, if the maternal rash happens more than 5 days before delivery, there is sufficient maternal anti-VZV IgG production, allowing transplacental transfer to the fetus. This passive immunity helps protect the newborn, resulting in a milder form of chicken pox [4].

Affected newborns with neonatal varicella typically present with fever, followed by a generalized vesicular eruption, which may be accompanied by listlessness and decreased appetite (anorexia). The rash initially appears as red macules, progressing to vesicles and encrustation, often causing pruritus (itchiness). The lesions typically first appear on the head before spreading to other parts of the body and may be observed in various stages of development and healing. The generalized distribution and presence of lesions at different developmental stages help distinguish varicella from neonatal herpes simplex virus (HSV), which tends to present as localized vesicular clusters. In mild cases, neonatal varicella lesions heal within 7–10 days [1].

The two cases of neonates in our study presented with classic clinical features of varicella neonatal, including a generalized vesicular rash followed by fever within the first 10 to 12 days of life, without dermatological lesions or congenital malformations at birth. In both cases, maternal chicken pox developed within the first few days postpartum, suggesting postnatal transmission as the likely source of infection.

The management of neonatal varicella remains a subject of debate due to the unpredictable severity of the infection and the absence of a standardized treatment protocol. Regarding prophylaxis, the Centers for Disease Control and Prevention (CDC) and the Advisory Committee on Immunization Practices (ACIP) recommend intramuscular VZIG, as soon as possible during the first 10 days of life, to newborns whose mothers have developed symptoms of varicella from 5 days before to 2 days after delivery. This window is chosen because of the timing of maternal antibody development to varicella infection and resultant transfer to the fetus. The infant is the most vulnerable to severe disease during this window. Some experts also recommend administration of varicella-zoster Ig to term infants who have been exposed to varicella during the first 2 weeks after birth and whose mothers do not have evidence of immunity. The recommended dosage is 125 units/10 kg and 62.5 units in neonates with birth weights below or equal to 2 kg. Isolation from the mother is also recommended until she is no longer infectious. If the specific VZIG is not available, IVIG (immune globulin intravenous) could be used, with a dosage of 400 mg/kg [7,9,10].

The recommended treatment for neonatal varicella is intravenous acyclovir at a dosage of 30 mg/kg/day, divided into three doses per day for 7 to 10 days [4,11]. If the intravenous lines cannot be placed to give intravenous acyclovir, oral acyclovir may be used as an alternative, at a dosage of 60 mg/kg/day [12]. A central line should only be recommended in case of clinical worsening [11]. Antiviral therapy should be initiated as early as possible, ideally within the first 72 h after the appearance of skin lesions, because most viral replication has stopped after this period [3]. Although valacyclovir has been proposed as an alternative antiviral agent, its use in neonates remains poorly studied [13]. Broad intravenous antibiotics should be given in addition to acyclovir to cover superimposed bacterial infection and can be de-escalated to a narrow spectrum if a source is identified [12].

In our cases, the neonates were isolated from their mothers and managed in a separate isolation unit. Prophylaxis with varicella-zoster immunoglobulin (VZIG) or intravenous immunoglobulin (IVIG) was not administered due to limited availability in our setting. Both patients received intravenous acyclovir for seven days and were additionally treated with broad-spectrum antibiotics ampicillin–sulbactam. The combination of ampicillin and sulbactam has been approved by the U.S. Food and Drug Administration (FDA) for the treatment of secondary skin infections, including superinfection of vesicular lesions [14]. Similarly, a 2024 study from Cambodia reported a 15-day-old infant with neonatal varicella in a resource-limited setting. Vesicular lesions appeared on day 7 of life. After 10 days of oral acyclovir and 5 days of antibiotics, all lesions had crusted. Neither IVIG nor VZIG was given because the products were not accessible. The infant was discharged without neurological or respiratory complications [15]. Likewise, a 2025 study from Yemen described a 13-day-old infant with neonatal varicella. Vesicular lesions appeared on day 11 of life. The infant was successfully treated with a 7-day course of intravenous acyclovir alone, without VZIG, IVIG because these products due to their unavailability [16]. Considering our findings and the two comparable studies from similar resource-limited settings, we propose that neonatal varicella in resource-limited settings can be effectively managed with acyclovir-based antiviral therapy alone, obviating the need for IVIG and avoiding potentially delay-inducing referrals to higher-level centers.

While primary varicella infection is generally a common and self-limiting illness during childhood, serious complications and mortality may occur, particularly in newborns. Delayed administration of antiviral therapy is one of the key risk factor for complications because initiating treatment more than 72 h after rash onset provides minimal benefit, and viral replication has largely ceased by that time, so late therapy does not prevent serious complication [15]. Other predictors of severe outcomes include maternal rash occurring five days before to two days after delivery, which leaves the newborn without trans-placentally acquired antibodies, prematurity or low birth weight, both of which are associated with diminished innate immunity, and symptom onset on or before the fifth day of life, a marker of massive viremia developing before maternal antibodies can be transferred [3].

Among the various complications, varicella-zoster virus (VZV) pneumonia is well-recognized, occurring in 15–30% of neonates with varicella; if left untreated, it carries a reported mortality of 20–30% [3]. Despite the known association between VZV infection and pneumonia, respiratory complications in pediatric varicella cases remain limited. Nationwide prospective cohort studies conducted in England, Germany, and Brazil have reported that VZV pneumonia is associated with 7.3 to 12.7% of varicella-related hospitalizations in pediatric settings [17,18,19].

In our case, the neonate in Case 2 developed pneumonia and was initially treated with ampicillin–sulbactam and gentamicin. According to recent studies, in addition to its role in treating secondary skin infections, ampicillin combined with an aminoglycoside such as gentamicin is considered an appropriate starting regimen for neonatal pneumonia [20]. However, due to clinical worsening, ampicillin was discontinued and replaced with cefotaxime, which was administered for a total of 12 days. Recent studies also suggest that cephalosporins such as cefotaxime can serve as an alternative combination therapy for neonatal pneumonia [21]. In addition to antiviral and antibiotic therapy, appropriate fluid management is essential in neonatal varicella cases to ensure adequate hydration and metabolic support. In our study, the neonates received maintenance fluids at a rate of 150 mL/kg body weight per day. This approach aligns with the recommended fluid requirements for term neonates, which range from 120 to 150 mL/kg/day between days 5 and 28 of life [22]. We provide a table summarizing practical recommendations for clinicians managing neonatal varicella in resource-limited settings (Table 4).

## 5. Conclusions

Although varicella infection is a generally self-limiting disease in childhood, neonatal cases carry a significantly higher risk of severe complications and mortality. In resource-limited settings, such as Indonesia and other developing countries, where access to IVIG may be restricted, pediatricians should be encouraged to implement alternative treatment strategies. Early initiation of antiviral therapy, timely administration of antibiotics to prevent and treat secondary bacterial infections, comprehensive supportive care, and careful monitoring for potential complications play a crucial role in managing neonatal varicella. Our findings highlight that timely and well-structured management can significantly improve neonatal varicella outcomes, even without IVIG.

## Figures and Tables

**Table 1:** Blood laboratory tests results.

Examination	Day 1	Day 6	Reference Range	Unit
Hemoglobin	13.0	12.0	10–18	g/dL
Hematocrit	38.1	34.9	31.00–55.0	%
Erythrocytes	4.29	4.11	3.0–5.4	million/uL
Leukocytes	12.21	12.42	5.0–19.5	10^3^/uL
Platelets	223	654	150–450	10^3^/uL
C-Reactive Protein	0.2	0.08	<0.5	mg/dL
Procalcitonin	0.34	0.21	0.18–0.39	%
Random Blood Glucose	93	109	70–140	mg/dL

**Table 2:** Blood laboratory tests results.

Examination	Day 1	Day 6	Day 13	Reference Range	Unit
Hemoglobin	16.0	13.1	12.3	10–18	g/dL
Hematocrit	46.7	36.4	35	31–55	%
Erythrocytes	4.77	3.85	3.81	3.0–5.4	million/uL
Leukocytes	8.19	8.93	10.49	5.0–19.5	10^3^/uL
Platelets	342	129	494	150–450	10^3^/uL
C-Reactive Protein	0.81	0.5	0.17	<0.5	mg/dL
Procalcitonin	0.39	0.41	0.55	0.17–0.32	%
Random Blood Glucose	95	84	102	70–140	mg/dL

**Table 3:** The main symptoms of FVS.

No	Clinical Presentation
1.	Skin lesions (60–70%). Cicatricial scars and skin loss.
2.	Central nervous system defects or disease (60%). Microcephaly, seizures, encephalitis, cortical atrophy and spinal cord atrophy, intellectual disability, cerebral calcifications, and ventriculomegaly.
3.	Ocular abnormalities (60%). Microphthalmia, chorioretinitis, cataracts, optic atrophy, nystagmus, and Horner syndrome (ptosis, miosis, and enophthalmos).
4.	Limb abnormalities (50%), which often include hypoplasia of the bone and muscle or rudimentary digits.
5.	Prematurity and intrauterine growth restriction (35%).

**Table 4:** Practical recommendations for clinicians managing neonatal varicella in resource-limited settings.

Key Step	When to Start	First-Line Regimen and Dose	Duration/Monitoring
Antiviral therapy	Ideally ≤72 h after first vesicle Start immediately if maternal rash −5 d to +2 d from delivery	Acyclovir i.v. 10 mg/kg q8 h (total 30 mg/kg/d). If i.v. access impossible, Acyclovir p.o. 20 mg/kg q6 h (total 80 mg/kg/d), as an alternative	7–10 days. Stop once afebrile and all lesions crusted.
Broad-spectrum antibiotics	If needed, to cover superimposed bacterial infection	Ampicillin–sulbactam 50 mg/kg (ampicillin component) q12 h and gentamicin 4–5 mg/kg q24 h	Reassess at 48 h. De-escalate to narrow agent if organism isolated. Total 5–7 days.
Supportive fluid and nutrition	Begin on admission	Maintenance fluids 120–150 mL/kg/d	Once the infant can meet most of its fluid needs orally, intravenous fluids should be tapered by 20–30 mL/kg every 12–24 h until discontinued.

## Data Availability

The original contributions presented in this study are included in the article. Further inquiries can be directed to the corresponsing author.
